# Supplementary material for: Shifts in the selectivity filter dynamics cause modal gating in K+ channels
Source: Nat Commun. 2019 Jan 10;10:123. doi: 10.1038/s41467-018-07973-6 (PMC6328603; doi:10.1038/s41467-018-07973-6)
Supplement: Supplementary file 2 — Supplementary Information [file 41467_2018_7973_MOESM2_ESM.pdf]

Supplementary Information

Shifts in the selectivity filter dynamics cause modal gating in K^+ channels

S. Jekhmane, J. Medeiros-Silva et al.

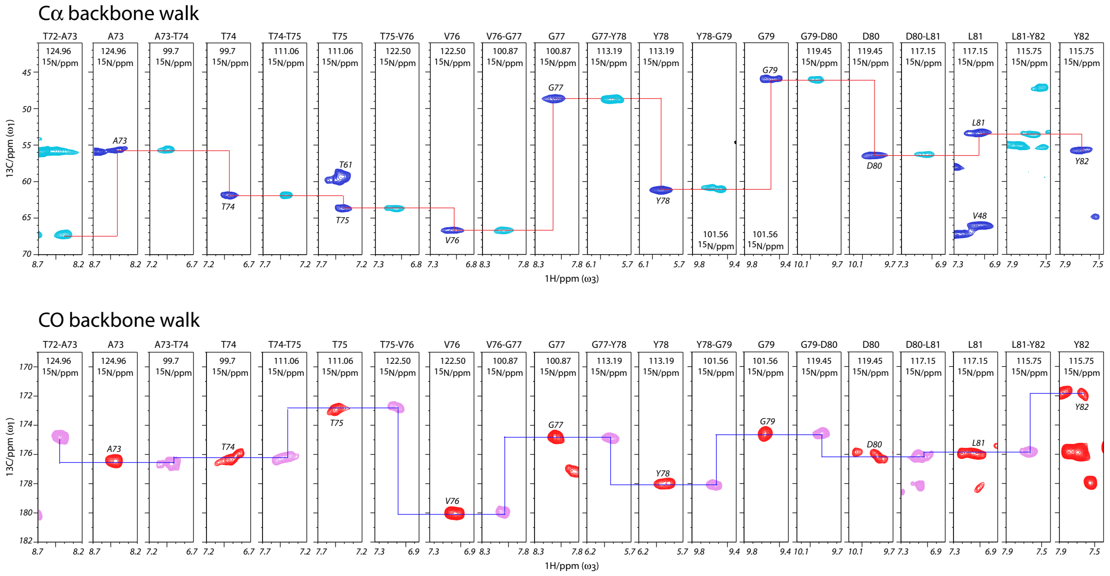

Supplementary Figure 1

Sequential ssNMR assignments with ^{1}H-detected 3D experiments

Upper panel: Cα-Cα^{+1} backbone walk showing full connectivity for residues T72 – Y82 in KcsA mutant E71A. Dark Blue signals show CAH planes from a 3D CANH experiment, cyan CAH planes were taken from a 3D CAcoNH experiment. *Lower panel:* CO^{-1}-CO backbone walk showing full connectivity for residues T72 – Y82 in E71A. Magenta signals show COH planes from a 3D CONH experiment, red COH planes were taken from a 3D COcaNH experiment.

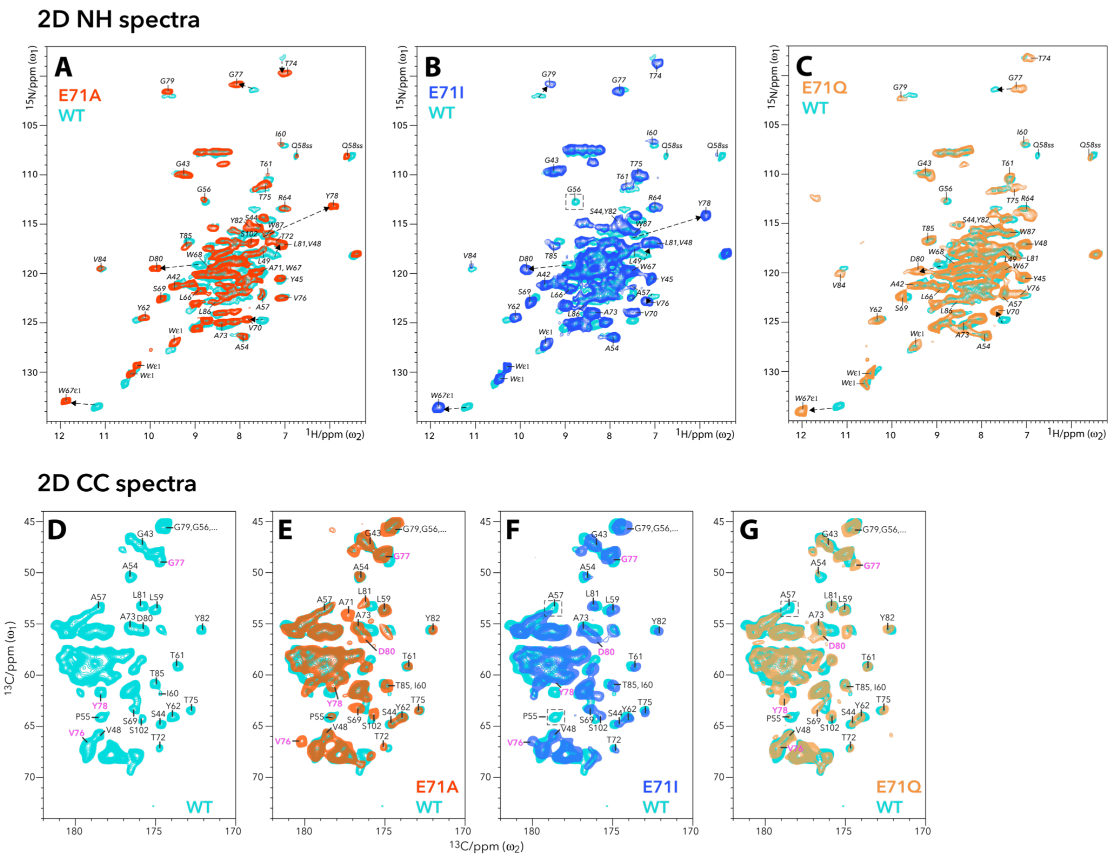

Supplementary Figure 2

Comparison of 2D NH and 2D CC ssNMR spectra of WT KcsA and the E71X mutants

Upper row: Overlay of 2D NH spectra of WT KcsA (cyan) onto A) E71A (red), B) E71I (blue), and C) E71Q (orange). *Lower row:* Zoom into the carbonyl-region of 2D PARISxy[1] CC experiments. D) WT KcsA (cyan), E) E71A (red) and WT KcsA, F) E71I (blue) and WT KcsA, G) E71Q (orange) and WT KcsA. Signals of V76-Y78 and D80 that show larger chemical shift perturbations are highlighted in magenta. Signals of P55-A57 that disappear or split in E71I due to conformational heterogeneity are highlighted in dashed boxes.

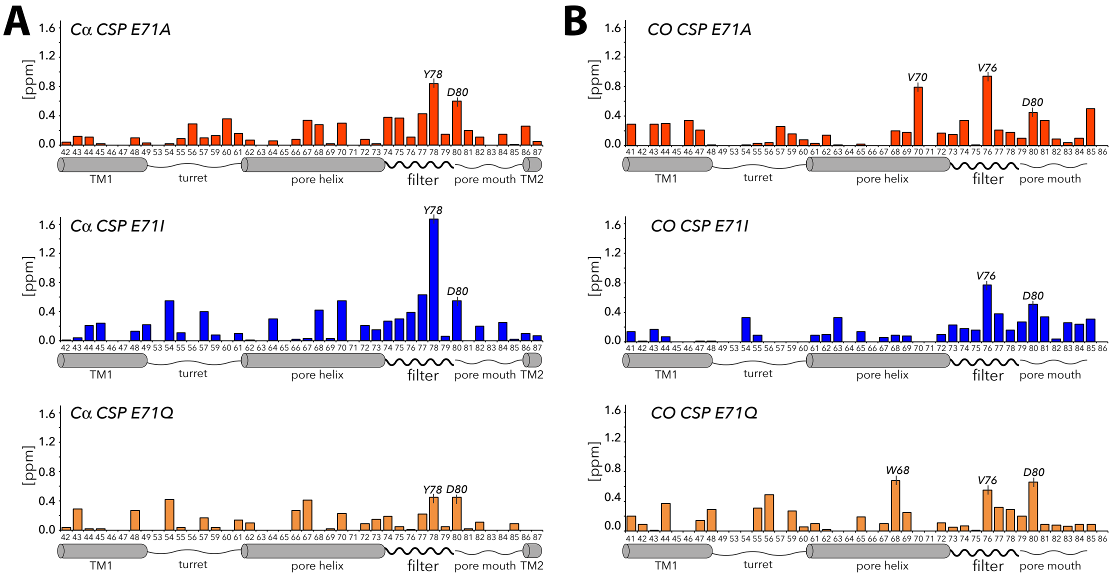

Supplementary Figure 3

Ca and CO ssNMR chemical shift perturbations of the E71X KcsA mutants

Left panel: Cα CSPs for E71A (in red), E71I (blue), and E71Q (orange). *Right panel:* Corresponding CO CSPs. Source data are provided as a Source Data file.

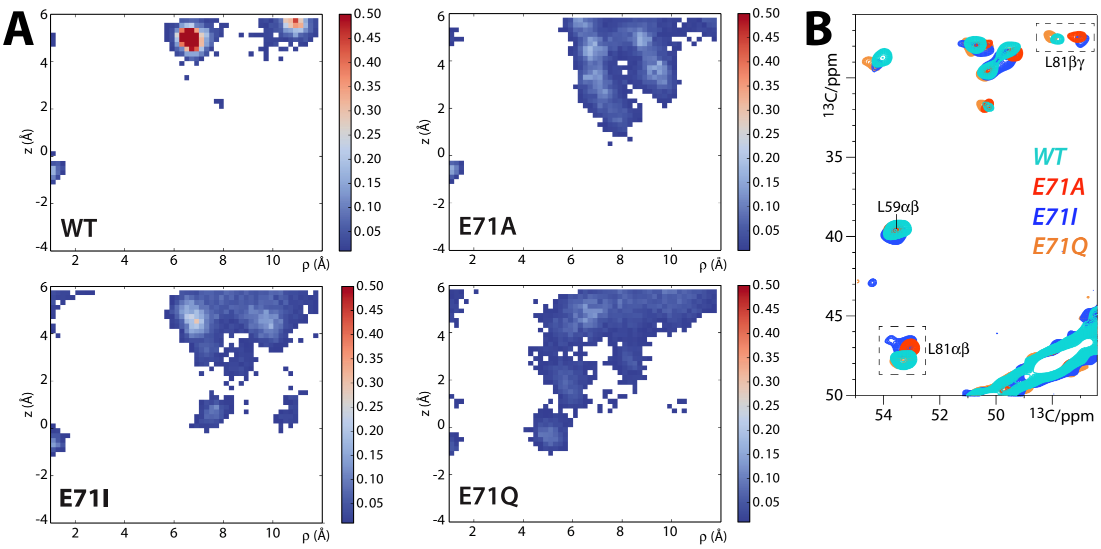

Supplementary Figure 4

Water distribution derived from long MD simulations / Structural changes in the water-lid

A) 2D average occupancy map for all four subunits during 1000ns MD simulations. The x-axis describes the radius to the center of the selectivity filter, and the y-axis is the z-coordinate of water molecules. In agreement with our ssNMR data, MD simulations show a widening of the water cavity for mutant channels E71I and especially E71A. In these channels, the cavity water also exhibits a higher mobility, as described by higher exchange-rates with bulk water (see C). For E71Q, the simulations could not reproduce the smaller water cavity that we unambiguously detect in solid-state NMR experiments. Potential reasons for the mismatch between ssNMR and MD simulations for E71Q could be a suboptimal MD starting structure (which was derived from WT KcsA) and insufficient sampling. B) The increased exchange with bulk water in mutants E71A and E71I most likely relates to structural and dynamical changes of residues D80 – Y82, which act as lid of the water cavity.[2] Indeed, we observe marked structural changes in the L81 side-chain conformation in 2D CC ssNMR spectra of mutants E71A and E71I.

C)

MD derived water exchange rates with bulk water

	WT	E71I	E71A	E71Q
# of water molecules	0.81±0.12	1.47±0.21	2.30±0.16	1.58±0.16
Turnover time (ns)	51.9±40.9	7.51±4.82	18.5±8.9	6.7±1.9

The water-binding cavity within each subunit is defined as the space with a distance less than 7 A° from both the nitrogen (or substituted ester oxygen) atom from Gly77 and the C-alpha from E71 in the same monomer. The number of water molecules within each subunit was determined as the average water number within this pocket during specific trajectory. The mean turnover time of water molecules within each subunit is defined as T_MD <N> = n_d, where T_MD is the length of the trajectory, <N> is the average number of water molecules in one cavity, and n_d is the number of distinct water molecules that visited the cavity at least once during the simulation. Each of the four subunits was treated as an individual sample to estimate the average and SD. This kind of analysis was also used in previous studies.[3]

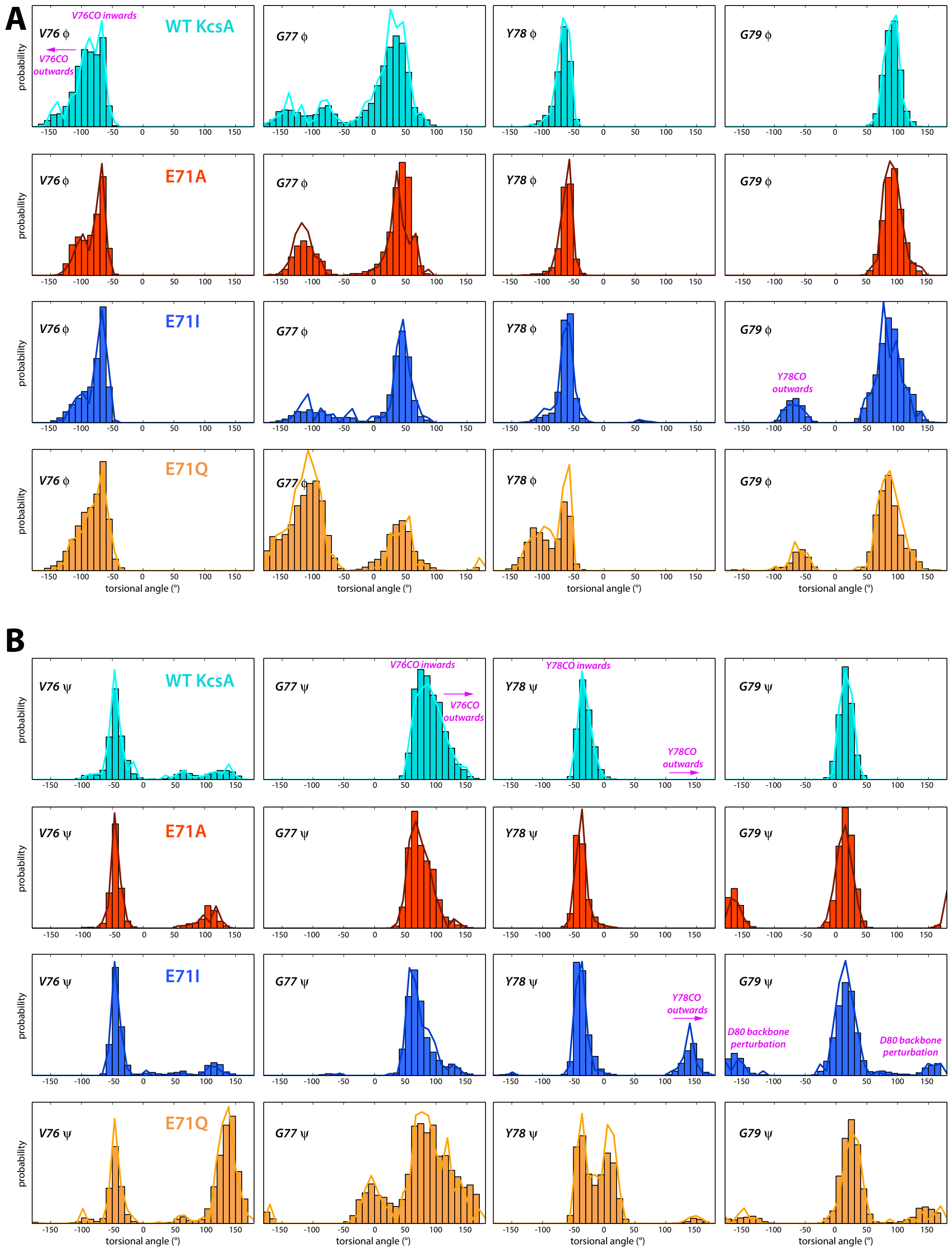

Supplementary Figure 5

Simulated dihedral angle profiles of WT KcsA and the E71X mutant channels

A) Phi (ϕ) and B) Psi (ψ) torsional angle distribution of selectivity filter residues of WT KcsA (cyan), E71A (red), E71I (blue), and E71Q (orange) derived from 1 μs-long MD simulations.

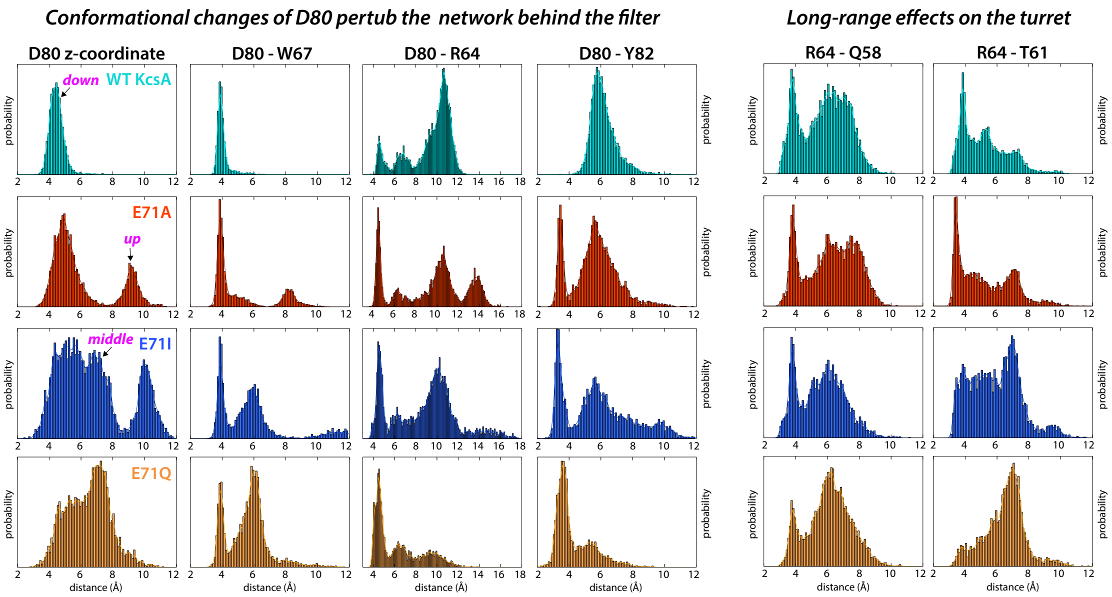

Supplementary Figure 6

Distance measurement between side chains derived from MD simulations

Distance measurement between side chains derived from 1 μs-long MD simulations of WT KcsA (cyan), E71A (red), E71I (blue), and E71Q (orange). The D80 side chain conformation is shown as the distribution of its z-coordinate (the centre of mass of carboxyl group). For distance measurement, the atom selections are D80 (Cγ), Y82 (OH), W67 (Nε1), T61 (Oγ1), Q58 (backbone carbonyl oxygen), and R64 (Cζ). Q58, T61, R64, W67, and D80 are from the same subunit, while Y82 is from an adjacent subunit.

References

1. Weingarth, M., Bodenhausen, G. & Tekely, P. Broadband magnetization transfer using moderate radio-frequency fields for NMR with very high static fields and spinning speeds. *Chem Phys Lett* **488**, 10-16, doi:10.1016/j.cplett.2010.01.072 (2010).
2. Ostmeyer, J., Chakrapani, S., Pan, A. C., Perozo, E. & Roux, B. Recovery from slow inactivation in K+ channels is controlled by water molecules. *Nature* **501**, 121-124, doi:10.1038/nature12395 (2013).
3. Li, J. *et al.* Chemical substitutions in the selectivity filter of potassium channels do not rule out constricted-like conformations for C-type inactivation. *P Natl Acad Sci USA* **114**, 11145-11150, doi:10.1073/pnas.1706983114 (2017).
